# The complete mitochondrial genome sequence of snake mackerels *Paradiplospinus antarcticus* (Scombroidei, Gempylidae)

**DOI:** 10.1080/23802359.2020.1775506

**Published:** 2020-06-08

**Authors:** Zhengbao Li, Jiwei Qi, Quan Ran, Yucheng Xu, Han Yu, Pengxiang Xu, Jie Zhang

**Affiliations:** a Anhui Key Laboratory of Eco-engineering and Bio-technique, School of Life Sciences, Anhui University, Hefei, China; b Key Laboratory of Animal Ecology and Conservation Biology, Institute of Zoology, Chinese Academy of Sciences, Beijing, China; c LiaoningPelagic Fisheries Co., Ltd, Dalian, China

**Keywords:** *Paradiplospinus antarcticus*, mitochondrial genome, snake mackerel

## Abstract

For the first time, we illuminate the complete mitochondrial genome (mitogenome) sequence of the *Paradiplospinus antarcticus,* which is 16,988 bp in size and contains 13 protein-coding (PCGs), 2 rRNA genes, 22 tRNA genes, and one control region.The base composition of the mitogenome is 26.08% A, 26.77% T, 28.46% C and 18.69% G. Here, we selected 11 genera of species from the mostly monotypic snake mackerel family, including representative Antarctic *Paradiplospinus antarcticus* that have been identified, and constructed phylogenetic trees to better study the snake mackerel family.

Fishes of the family Gempylidae (snake mackerels) are widely distributed in deeper tropical and subtropical waters (Nakamura and Parin 1993). There are 24 species in 16 genera in the world (Nelson et al. 2016). However, the question of the classification status of the snake mackerel family in the Scombroidei has been puzzling for some time (Johnson 1986).One of the snake mackerels, *Paradiplospinus antarcticus,* was discovered and named by Andriashev in 1960 (Andriashev 1960). *Paradiplospinus antarcticus* and *Paradiplospinus gracilis* are similar in morphology, with some overlap in distribution. Some species collected from the Southern Ocean are directly referred to as ‘*Paradiplospinus gracilis*’, and it is controversial whether the two are the same.

The valuable samples (Deposit Number: Fish-1-Antarctic 36) were collected in the Southern Ocean (63°01′S, 058°05′W) and stored in the Fish Collection in the Institute of Zoology, Chinese Academy of Sciences, Beijing, China (IOZCAS). Total genomic DNA was isolated using Easy Pure@Genomic DNA Kit according to the manufacturer’s instructions (TransGenBiotech Co, Beijing, China). The mitochondrial genome sequence of *Paradiplospinus antarcticus* was determined and annotated by Biomarker Technologies Co, LTD, Beijing, China. Phylogenetic trees were reconstructed based on the 13protein-coding genes (PCGs) of 12 snake mackerels and out-group taxa using IQ-TREE web server with the TPM2 + F + I + G4 model (Trifinopoulos et al. 2016).

The complete and circular mitochondrial genome is 16,988 bp in size, it has been deposited in NCBI and Genbank (accession: MN510443) with an AT bias of 52.85%, and includes 13 protein-coding genes (PCGs), 22 transfer RNA genes (tRNA), 2 ribosomal RNA genes (12S rRNA and 16S rRNA), and one control regions (CR). There are ND6 subunit gene and eight tRNAs on the L-strand, and the other 28 genes on the H-strand. Thirteen PCGs are 12,869 bp and start with typical ATN codons except COX1 with GTG; The TAG was found as the stop codon in COX1, ND3, ND5 and ND6, the TAA was found as the stop codon in ND1, ND2, ATP8, ATP6, COX3 and ND4L, while the incomplete codon T was found as the stop codon in COX2, ND4 and CYTB; The 22 tRNA genes range in size from 66 bp in tRNA^Cys^ to 75 bp in tRNA^Leu^ and tRNA ^Lys^, the12S and 16S rRNA are 956 and 1687 bp, respectively. The result showed that most of the nodes in the tree graph have higher SH-aLRT and ultrafast bootstrap values (Figure 1). Besides, *Paradiplospinusantarcticus* and *Diplospinus multistriatus,* are closely clustered to support previous results based on morphological studies (Jondeung and Karinthanyakit 2010).

**Figure 1.**
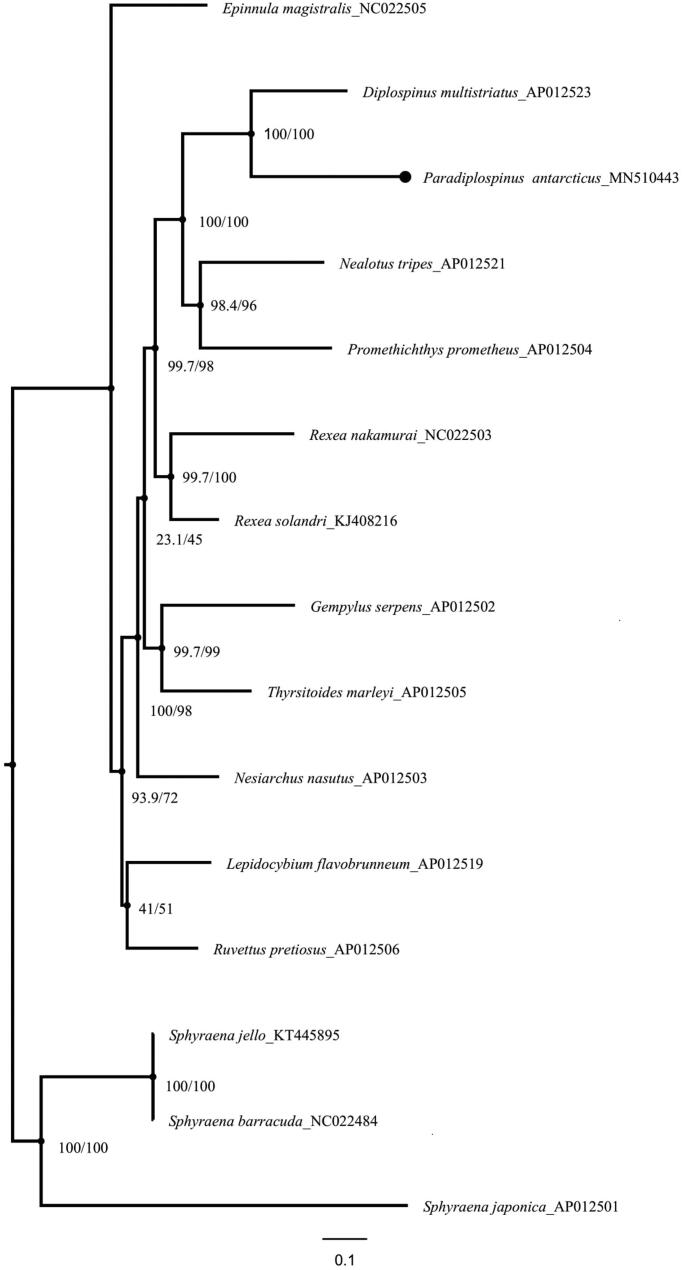
Phylogram showing the phylogenetic relationship of Scombroidei based on themitogenome. The values on nodes indicate the results of SH-aLRT and ultrafast bootstrap tests with 10,000 replicates, respectively.

## Data Availability

The data that support the findings of this study are openly available in [NCBI], reference number [https://www.ncbi.nlm.nih.gov/nuccore/MN510443.1/].
